# DNA甲基化在肺癌中的研究进展

**DOI:** 10.3779/j.issn.1009-3419.2010.11.15

**Published:** 2010-11-20

**Affiliations:** 200433 上海，同济大学附属上海市肺科医院胸外科 Department of Thoracic Surgery, Shanghai Pulmonary Hospital Affiliated to Tongji University, Shanghai 200433, China

目前，肺癌仍为人类因癌症死亡的主要原因^[1]^，全世界每年有150万人死于肺癌。临床上肺癌患者就诊时大多已属中晚期，错失手术机会，5年生存率不足15%^[2]^，肺癌如此高的死亡率与缺乏早期检测手段及欠佳的治疗措施有关，要想提高生存率，只有通过早期发现、早期诊断和早期手术治疗等来实现。目前虽然对肺癌形成及发生发展机制已进行了大量研究与探索，但其确切机制尚未明确。许多研究^[3-5]^表明，DNA甲基化与多种肿瘤的发生发展密切相关，是肿瘤发生发展的重要机制之一。在肺癌发生发展过程中，DNA甲基化修饰异常也扮演着重要角色。了解DNA甲基化修饰异常在肺癌发生发展中的机制，将有助于肺癌患者的早期发现、早期诊断、早期治疗并改善预后。本文就DNA甲基化修饰异常在肺癌中的研究进展综述如下。

## 1 DNA甲基化

DNA甲基化是基因表观遗传学修饰方式之一，可能存在所有高等生物中。在哺乳动物体内，DNA甲基化是指生物体在DNA甲基转移酶（DNA methyltransferase, DNMTs）的催化下，以S-腺苷甲硫氨酸为甲基供体将胞嘧啶核苷酸（C）第5位碳原子甲基化变为5’-甲基胞嘧啶（5’-mC）的一种反应。而甲基胞嘧啶可以脱氨基转化成胸腺嘧啶（T），造成C-T突变进而导致某些基因突变而失活，这个过程调控了基因的表达。DNA甲基化常发生于CG二核苷酸密集区，这些区域被称为CpG岛，健康人基因组中，CpG岛中的CpG位点通常是处于非甲基化状态，而CpG岛外的CpG位点则通常是甲基化的，这种甲基化的存在形式在细胞分裂过程中能够稳定保留^[6]^，并可以遗传，是维持高度有序的染色质结构的重要因素。当一些抑癌基因CpG岛中的CpG序列呈高甲基化状态时，其可致染色体螺旋程度增加及抑癌基因沉默和表达缺失^[7]^，引发肿瘤生长。DNMTs在整个初始甲基化及甲基化的维持中发挥着重要作用，目前认为DNMTs主要包括DNMTl、DNMT2、DNMT3a和DNMT3b共4个成员，其中DNMT1负责准确复制DNA甲基化形式，起着维持甲基化的作用，DNMT2最初被认为是一种DNA甲基化转移酶^[8]^，但是最近研究^[9]^发现其可以调节tRNA的甲基化，DNMT3a和DNMT3b被认为是从头甲基化转移酶，主要负责非甲基化CpG位点的甲基化。DNA甲基化对基因表达的影响需要一些甲基化的CG序列结合蛋白（methyl-CpG-binding domain protein, MBDs）共同参与^[10]^，这些活性蛋白通过恢复组蛋白修饰和染色质重构酶作用促使染色质纤维由10 nm的疏松结构转变为一个浓聚的30 nm的螺旋结构而发挥作用。DNA甲基化状态可受感染、环境以及营养因素的影响，但其机制还不清楚，可能是这些因素能影响DNA甲基化转移酶的合成、活性和作用靶点，或者影响MBDs和脱甲基酶。

## 2 DNA甲基化与肺癌

到目前为止，在肺癌患者中已检测出大量基因发生甲基化，大部分研究是基于外科手术获得的非小细胞肺癌样本。目前非小细胞肺癌样本中集中研究的基因包括*p16*、*RASSF1A*、*CDH1*、*CDH13*、*APC*、*RARβ*、*DAPK*和*MGMT*^[11]^。其中*p16*基因位于人第9条染色体p21区，参与细胞周期蛋白调控，通过与细胞周期蛋白依赖激酶CDK4及CDK6结合而抑制后者活性，进而抑制细胞增殖，是一种重要的抑癌基因，其失活与多种肿瘤的发生发展密切相关，p16启动子区5’-CpG岛甲基化是其失活的重要原因，在肺癌发生发展中起重要作用。肺癌中*p16*基因甲基化频率各学者报道不同，在肺癌组织中甲基化发生率大多在17%-80%，在肺癌患者血液中其发生率大多在13%-77.8%；Ras相关区域家族1A（RASSF1A）是人类肿瘤中甲基化频率最高的一个抑癌基因，RASSF1A主要参与细胞周期的调节，该基因甲基化与肺癌发生发展及预后关系密切。综合近几年报道，RASSF1A在原发非小细胞肺癌中甲基化率在30%-40%，在小细胞肺癌中甲基化率几乎可以达到80%^[12]^。Tomizawa等^[13]^报道110例Ⅰ期肺腺癌患者*RASSF1A*甲基化率为35%，且分化差的肿瘤比高分化和中分化的肿瘤甲基化频率高。最近张卉等^[14]^报道，非小细胞肺癌组织中RASSF1A启动子甲基化率为38.7%，20例肺良性病变中无一例发现RASSF1A启动子甲基化。Wang^[15]^研究发现deltaDNMT3B4的表达与RASSF1A启动子甲基化状态密切相关，他们通过RNA干扰技术敲除非小细胞肺癌细胞株中的deltaDNMT3B4而导致RASSF1A启动子去甲基化而复活；*CDH1*、*CDH13*基因均属于钙粘蛋白家族，CDH1编码的蛋白E-cadherin是一跨膜糖蛋白，在肿瘤侵袭转移方面起重要作用，是公认的浸润转移抑制基因。该基因启动子区CpG岛甲基化是E-cadherin失活的重要机制，目前报道的非小细胞肺癌中*CDH1*基因启动子CpG岛甲基化率在15%-46%。王红兵等^[16]^最近报道在肺癌组织中*CDH1*基因甲基化率为40.9%，明显高于癌旁组织和正常组织，提示CDH1启动子CpG岛甲基化可能参与肺癌的发生发展。*CDH13*基因是钙粘家族的又一成员，编码蛋白H-cadherin，起着抑癌基因作用，启动子区CpG岛甲基化是H-cadherin失活的重要机制；APC可抑制β-catenin，是家族性或散发性结肠癌相关抑癌基因，其启动子因甲基化而失活在肺癌发生发展中的重要作用也被证实。Brabender^[17]^报道在非小细胞肺癌组织中*APC*甲基化率高达94%，而正常对照组仅20%发生甲基化。RAR-β主要调节细胞生长过程。Virmani等^[18]^报道*RAR-β*基因甲基化率为28%，且与肿瘤细胞分化程度密切相关，在非小细胞肺癌患者的预后方面具有临床参考价值；死亡相关蛋白激酶*DAPK*基因作为抑癌基因具有调节凋亡的功能，与肺癌的发生发展密切相关，其因启动子甲基化而表达缺失可导致多种肿瘤发生。O_6_-甲基鸟嘌呤-DNA-甲基转移酶（O_6_-methylguanine-DNA methyltransferase, MGMT）是一种高效修复酶，能修复DNA序列中6-氧-甲基鸟嘌呤损伤，对维持基因组的稳定性有重要意义，该基因的表达缺失可以导致肺癌的发生发展，其启动子区甲基化是MGMT去表达的重要机制。Esteller等^[19]^报道非小细胞肺癌组织标本中*MGMT*甲基化发生率为29%。孔云明等^[20]^报道肺癌患者血浆标本中*MGMT*甲基化率为24.62%。研究^[21]^发现MGMT启动子甲基化可能与*p53*突变相关。另外，与非小细胞肺癌相关的甲基化基因还有：*FHIT*、*HIC-1*、*AKAPl2*、*ESRl*、*CYGB*、*OPCML*、*ADAMTSl*、*TGFBI*、*RUNX3*、*UMDl*、*hSRBC*、*CADM1*、*p14ARF*、*p16INK4a*、*DAPK*、*GSTP1*、*MGMT*、*MLH1*、*FBN2*、*DAL-1*、*ASC*等^[22-28]^。

最新发现的几个肺癌相关基因甲基化如下：Zhang等^[29]^研究了78对非小细胞肺癌组织、癌旁组织及25个良性病变组织发现*DLEC1*基因甲基化率分别为41%、3.8%和0，*DLEC1*甲基化程度与肺癌分级及淋巴结转移情况相关。他们还检测了肺癌患者血浆样本中*DLEC1*甲基化率为35.9%，而非癌患者血浆中该基因甲基化率仅为2%，DLEC1在血浆中甲基化状态和组织中一致性较好。*DLEC1*基因启动子甲基化在肺癌组织中表达沉默，而在癌旁和良性病变组织中则广泛表达。Nagji等^[30]^研究发现乳腺癌转移抑制基因1（breast cancer metastasis suppressor 1, BRMS1）的mRNA和蛋白表达在非小细胞肺癌中明显降低，他们发现非小细胞肺癌样本BRMS1启动子甲基化率明显高于癌旁组织，在鳞癌中该差别更明显。BRMS1启动子甲基化与临床病例特征联系分析得知在鳞癌中BRMS1甲基化与病理分期相关。Suzuki等^[31]^检测了17个非小细胞肺癌细胞株和236个非小细胞肺癌组织标本中CXCL12的mRNA和甲基化状况发现，CXCL12在236个肺癌组织中甲基化率为36%，*CXCL12*甲基化状态和其表达显著相关。他们认为CXCL12沉默在非小细胞肺癌中是个频繁的事件。Chang等^[32]^研究发现非小细胞肺癌中RECK启动子甲基化率为63.6%，其表达下调与该基因启动子甲基化密切相关，*K-ras*基因的密码子12在肺癌组织中突变率为25.5%，统计分析显示*K-ras*突变与RECK启动子甲基化密切相关，他们认为在非小细胞肺癌中RECK下调是由于启动子甲基化引起的，且与*K-ras*突变和淋巴结转移相关。Yu等^[33]^报道，EPHB6启动子甲基化引起基因表达下调，在非小细胞肺癌侵袭转移中发挥重要作用，其高甲基化状态可增加非小细胞肺癌远处转移的风险。Rui等^[34]^发现，*FBLN-3*基因在非小细胞肺癌组织中甲基化率为43.1%，比相应正常组织甲基化率（9.2%）明显增高，且FBLN-3高甲基化与其mRNA和蛋白水平表达下调相关，研究还表明FBLN-3甲基化状态与肺癌分化程度、分期和淋巴结转移有关。

目前大多数甲基化研究大多集中在非小细胞肺癌中，而对小细胞肺癌的研究较少。Kreisler等^[35]^研究发现神经元抑制沉默因子/RE1沉默转录因子（neuron restrictive silencer factor or RE1-silencing transcription factor, NRSF/REST）是小细胞肺癌进展的一个重要调节剂，在小细胞肺癌中NRSF/REST本身起肿瘤抑制因子作用，NRSF/REST因自身甲基化和CREB调节而失活，NRSF/ REST的丢失导致表皮生长因子介导的AKT磷酸化，对细胞增生和生存有重要意义。Wang等^[36]^用Illumina Beadchip assay检测了44对小细胞肺癌和对照组外周血白细胞DNA的52个基因的62个CpG位点甲基化表达差异，并用pyrosequencing技术验证了9个CpG位点，统计分析甲基化差异发现这9个CpG位点甲基化预示一个较高的小细胞肺癌危险度，可能有助于小细胞肺癌的风险预测和诊断。他们发现CSF3R和ERCC1甲基化联合检查对区别小细胞肺癌和对照组意义较大。

## 3 DNA甲基化在肺癌中的应用

### 3.1 DNA甲基化在肺癌早期诊断中的应用

目前肺癌相关基因甲基化异常不仅在肺癌组织中被检测^[29-31]^，在肺癌患者的血清或血浆^[37, 38]^、痰^[39, 40]^、支气管刷检和活检标本^[41, 42]^中也能够检测到，最近Han等^[43]^认为DNA甲基化能够在呼出气的冷凝液中检测到。这些样本的最大好处是较易获得，在这些样本中检测DNA甲基化对肺癌早期检测及诊断具有潜在的用途。Licchesi等^[44]^对癌旁正常组织、肺腺瘤型增生过长组织（癌前病变）及相应的肺腺癌组织中7个基因甲基化进行研究，发现甲基化的频率从正常组织到肺腺瘤型增生过长组织（癌前病变）再到腺癌是明显增加的。这证明某些基因的甲基化可能是肺癌发展中的一个早期标志，且甲基化程度随恶性演变而增加。同时对几个基因的甲基化检测可能成为肺癌早期检测和风险评估的一个生物指标。

### 3.2 DNA甲基化在预测肺癌复发及预后中的应用

有研究发现DNA甲基化与肺癌的复发情况相关，Brock等^[45]^研究了Ⅰ期非小细胞肺癌术后血中DNA甲基化与肺癌复发情况的关系发现在肺癌组织和非转移性淋巴结中p16、CDH13、RASSF1A和APC的甲基化状态与肺癌的复发情况相关，如果p16和CDH13在肿瘤组织和纵隔淋巴结中被检测是高甲基化状态，则其复发的优势比为15.5，作者认为这些基因在正常淋巴结中高甲基化提示可能存在显微镜无法检测到的微转移灶，某些基因的高甲基化可能使细胞具有转移扩步的潜性，对预测疾病的复发情况可能有意义。原发性非小细胞肺癌中某些基因的甲基化可作为其预后不良的一个指标。Tang等^[46]^报道DAPK甲基化状态与Ⅰ期非小细胞肺癌患者的生存密切相关，这些发现在后来也被Lu等^[47]^研究证实。Burbee^[48]^和张卉^[14]^报道*RASSF1A*基因甲基化可作为非小细胞肺癌预后不良的一个指标。Kim^[49]^以及Tomizawa^[13]^也报道*RASSF1A*甲基化可作为Ⅰ期肺腺癌预后不良的一个指标。Toyooka等^[50]^报道*p16*的甲基化状态与肺腺癌的预后不良显著相关。在Ⅰ期非小细胞肺癌中，Yanagawa^[51]^发现*RASSF1A*和*RUNX3*甲基化与预后不良有关。Brabender^[17]^报道*APC*基因启动子区高甲基化与患者低生存率相关。Seng等^[52]^研究也发现*DLEC1*甲基化在原发性非小细胞肺癌中（包括鳞癌）是一个独立的预后指标，*hMLH1*甲基化是影响大细胞肺癌预后的一个指标。Nagji等^[30]^认为BRMS1是非小细胞肺癌一个预后不良的指标。Suzuki等^[31]^分析显示*CXCL12*甲基化与非小细胞肺癌患者预后密切相关，对CXCL12的甲基化分析可能成为非小细胞肺癌患者术后预后指标。

### 3.3 DNA甲基化的逆转在肺癌治疗中的应用

基于DNA甲基化导致抑癌基因沉默促使肿瘤发生的机理，用能够逆转甲基化的药物改变沉默基因的甲基化状态是目前一直在研究的用来治疗肺癌的一种手段。过去几年里，逆转基因甲基化改变的方法或药物已有报道，主要包括腺苷类DNA甲基化转移酶抑制剂和非腺苷类DNA甲基化转移酶抑制剂。5-氮杂胞苷和5-氮-2'-脱氧胞苷作为腺苷类DNMT抑制剂通过共价键与DNMT形成稳定的中间体而使DNMT失活，导致DNA去甲基化和沉默基因重新表达。目前该类药物主要用于血液系统肿瘤的治疗。非腺苷类DNA甲基化转移酶抑制剂主要是抑制DNMT活性，其主要包括EGCG（一种多酚化合物）、MG98（一种反义寡核苷酸）、普鲁卡因、普鲁卡因胺和肼屈嗪等。此外，还有些去甲基化的研究如：小分子干扰RNA（small interfering RNAs, siRNA）通过与DNMT mRNA结合导致其降解和基因沉默，从而使DNA发生去甲基化^[53]^。这些研究有望为肺癌治疗提供一种新方法。

## 4 结语

DNA异常甲基化与肺癌的发生发展密切相关，肺癌在其发生与发展过程中存在一系列基因异常甲基化。通过检测多个基因甲基化情况可对肺癌进行早期检测、诊断和判断复发及预后情况，同时了解DNA甲基化修饰异常在肺癌发生发展中的机制，可以用去甲基化药物使甲基化沉默基因重新表达，有望为肺癌的临床治疗提供新方法。然而其在肺癌诊断及治疗中的应用还存在一些问题，如目前甲基化研究多基于肿瘤组织，虽在血浆、痰和其它样本中也可检测到，但其灵敏性和特异性不如肿瘤组织，且在血液中不一定有足够能检测到的肿瘤DNA，特别在肿瘤的早期阶段，还有血液中的肿瘤标记物没有器官特异性，不能判断一定是肺癌；痰标本在吸烟人群较易获得，但在不吸烟人群不易获得^[11]^，且来自肺中央的痰标本不一定能够检测出周围型肺癌。另外，虽同时对多个基因甲基化检测比检测一个基因对肺癌诊断的敏感性和特异性高，但是哪一组基因甲基化有较高灵敏度和特异性，这些都是肺癌诊断中存在的问题。基因去甲基化治疗肿瘤也存在一些问题，如去甲基化药物虽可以改变DNA甲基化模式，但由于其可逆性的特征，同样有可能恢复到原始的甲基化状态，且长时间大剂量给予甲基化抑制剂可能产生严重的毒副作用，如恶心、呕吐、骨髓抑制、甚至基因突变导致其它肿瘤发生等。因此对于晚期肺癌患者，是否将去甲基化药物用于肿瘤治疗还有待进一步研究。
